# The affective assemblage of internationalisation in Japanese higher education

**DOI:** 10.1007/s10734-020-00593-4

**Published:** 2020-08-04

**Authors:** Louise Morley, Paul Roberts, Hiroshi Ota

**Affiliations:** 1 Centre for Higher Education and Equity Research (CHEER), University of Sussex, Falmer, Brighton, East Sussex, UK; 2 Hitotsubashi University, Kunitachi-shi, Tokyo 186-8601 Japan

**Keywords:** Japanese higher education, Affect, Mobility, Internationalisation

## Abstract

Positive attributes stick to higher education internationalisation, and it is a policy paradigm with performative effects. Internationalisation draws on imagined virtuous flows of knowledge production and exchange, and is presented as an assemblage of detraditionalisation, expansiveness and epistemic and cultural opportunity for individuals, organisations and nation states. Policies target bodies, minds and affect, yet are presented as an unquestionable good in an imagined genderneutral, borderless, meritocratic and benign global knowledge economy. This paper explores the affective economy of internationalisation drawing upon interview data gathered in fifteen private, five national and eight public universities in Japan with thirty-four migrant academics and thirteen international doctoral researchers. We aim to contribute to internationalisation theory by exploring the sticky micropolitics of internationalisation in relation to affective assemblages, and how the gendered, racialised, linguistic and epistemic inequalities constituting academic mobility are frequently disqualified from discourse. Our discussion includes consideration of the Japanese policy context, the concept of affective assemblages, navigating gender regimes, precarity and linguistic imperialism. We conclude that the immaterial or affective labour that is required to unstick, install and maintain an internationalised academic identity and navigate the translations and antagonisms from everyday encounters with difference is substantially under-estimated.

## Academic mobility: material, intellectual and affective transitions

Positive attributes have stuck to internationalisation, and it has become a policy paradigm with performative effects. Mobility is represented as a pleasurable formation while simultaneously framing migrant academics as human capital to attract international students, marketing enhancers and disposable labour. Similarly, international doctoral researchers are constructed in terms of income-generation, and indicators of internationalised knowledge networks (Lomer 2017; Owens et al. 2011). The social and intellectual benefits of multiculturalism, prejudice reduction, knowledge internationalisation, cosmopolitanism and soft power are blended with the economic and material benefits of global competitiveness, prestige, enhanced employability and elite research concentration. The university is a regulated affective space, with actors having both a force to affect and be affected. Internationalisation is performative in so far as it requires and produces a range of affects. In the neoliberal economic policy framing, the ideal internationalised subject is presented as a neutral category—unburdened by embodiment, social difference or affect. We aim to contribute to internationalisation theory by exploring the micropolitics of internationalisation and how the gendered, racialised, linguistic, epistemic and affective inequalities constituting academic mobility are frequently disqualified from discourse (Fahey and Kenway 2010). Borders, boundaries and spatialities are social and affective as well as material constructions, which extend beyond the notion of physical space (Anthias 2012). Internationalising oneself represents a cut in the continuity of the known. Theoretically, we aim to become analytically sensitive to affectivity, embody the abstract dimensions of mobility and explore the relationality between internationalisation and equity.

The absence of a theory of affect in internationalisation is surprising given that affective life has been subject to copious explanations and descriptions in the humanities and social sciences (Anderson 2009; Gregg and Seigworth 2010; Wetherell 2012). Internationalisation is a process that occurs in diverse sites, practices and relationships. People have often been nudged, rather than directed to internationalise, or unstick themselves from national locations, and it has increasingly become part of the ‘choice architecture’ for academics and doctoral researchers (Whitehead et al. 2014). Academia incorporates overt and subliminal levels of persuasion to internationalise one’s self, with movement prioritised over stasis. Cognitive capitalism values co-creativity, collaboration, co-knowing, and knowledge exchange across national boundaries (Ackers 2010; Dowling et al. 2007; Veijola and Jokinen 2018). Mobility demands literal and figural movement, unsettling the geo-affective subject and producing new assemblages. An assemblage is a multiplicity of interacting forces and components involving fluidity, exchangeability and multiple functionalities (Deleuze and Guattari 2008; Nail 2017). The assemblage of internationalisation can include an underbelly or shadow relating to market values, male, colonial and ethnic dominance, commodification and disposability of academic labour, linguistic imperialism and knowledge capitalism (Morley et al. 2018, 2019). Unsticking oneself, or becoming a fluid subject, can produce affective assemblages relating to belonging, inclusion/exclusion and difference. Transitions, adaptations and ruptures to the known are felt. Repetitions are disrupted, and difference is intensified, producing opportunities and frictions. There are generative moments of experience in moveable environments of potential and constraint. Massumi (2009) argues that affect is linked to movement, change and transmission—a type of ‘micro-shock’. While internationalisation provides conditions of possibility, it also abounds with affective intensities and resonances. It catalyses a range of micro-shocks that are linked to the psycho-social of space, place, difference and identity. The proliferation of differentiation involved in academic mobility can result in a potent relationship between affect, change and capacitation/decapacitation (Bjerg 2013). Certain subject positions are annulled, disqualified and evacuated of intelligibility, while others are elevated, augmented and reinforced.

While many internationalised academics are animated and positive about their experiences, translation through displacement from one context to another can sometimes be a form of symbolic violence, with affective consequences (Bondi and Davidson 2011). We argue that the immaterial or affective labour (Bialostok and Aronson 2016; Oksala 2016) that is required to unstick, install and maintain an internationalised academic identity and navigate the translations and antagonisms from everyday encounters with difference is substantially under-estimated. The paper proceeds in four directions. First, we examine the Japanese policy context; second, we explore the concept of affective assemblages; third, we describe the methods used to collect the empirical data for the research project; and fourth, we present three of the areas of affective intensity that emerged from our data- navigating gender regimes, precarity and linguistic imperialism.

## Japanese higher education

Japan is a society that is simultaneously intelligible and unintelligible—known and unknowable to foreigners, hence providing a rich setting for the study of affect. Japan is of particular interest as it has been portrayed in research as a country captivated by Shimaguni konjo (island nation mentality). Itoh (1998), Hall (1998) and Willis (2008) have all indicated that Japan has never recovered from the isolation of the Edo period. The *Kokusaika* or internationalisation of Japan’s higher education is now a policy priority (Agawa 2011; Goodman 2007; Mock et al. 2016; Rappleye and Vickers 2015; Rivers 2010), but ambiguously understood and applied. Strategies include the 2009 *Global 30* project and the 2014 *Top Global University Project* (Huang et al. 2019; MEXT 2014). Selected universities are required to recruit more international students and develop new English-medium instruction (EMI) degree programmes (Bradford and Brown 2017), and increase the proportions of international faculty. As with many countries, internationalisation in Japan is both a desired and feared force (Poole 2016), incorporating an awareness of symbolic goods such as recognition, distinction and competitive stratification in the prestige economy. The pressure of status competition for publishing, funding and ranking success and the power of vertical differentiation are structuring strategic rationalities that get stuck to national higher education systems. Contemporary internationalisation discursive practices relate to the desire for competitive participation in the global knowledge economy, and the creation of new geopolitical sites for world-class universities. Depending on the index, Japan had 3 (Academic Ranking of World Universities), 2 (Times Higher Education World University Rankings) and 5 (QS World University Rankings) universities in the top 100 league tables in 2019. Internationalisation plays a major part in aspirational leadership imaginaries to join the upper ranks of the global league tables.

Reform, in relation to accelerating status in the global knowledge economy, has been a key aspect of Japan’s higher education system (Amano and Poole 2005). There are aspects of Japanese higher education that interact with internationalisation e.g. excess supply over demand means that many universities that have been unable to meet their quotas are turning to the recruitment of international students. Japan has one of the largest higher education systems in the world comprising 786 universities, 326 junior colleges and 57 colleges of technology in 2019. It has a large private sector—approximately 78.6% (MEXT 2019), and three higher education sectors with different legal status (national, private and local public) (Kitagawa and Oba 2010). Japan’s national universities enrol 20% of students but receive 72% of the national higher education budget. There is an intense stratification and research concentration, with 15 universities receiving 50% of governmental research grants. Japan’s falling birth rate, ageing population and over-supply of university places mean that there is some urgency to diversify and internationalise (Rivers 2010). A view that stuck to the analysis of internationalisation in many of our participants’ narratives was that the numbers of migrant academics and international doctoral researchers were dominant performance indicators in the evaluation of the effectiveness of institutional policies. While multiple positive attributes could get stuck to them, as carriers of cosmopolitanism and linguistic privilege, for example, the socio-materialism of their own affective worlds was often overlooked.

## Affective assemblages

Internationalisation policy and process is formed by, and productive of affect. By affect, we mean emotions, responses, reactions and feelings that are construed as relational and transpersonal rather than located solely in the interior individual subject. Affects emerge from the dynamic relations of bodies and their interactions. They can be sticky as they are always a part of an encounter and always social in nature. Affect theory has the potential to facilitate the questioning, disruption, diagnosing and renewing of the cultures we inhabit and reproduce. Mazzarella (2009) argues that society is inscribed on our nervous system and in our flesh before it appears in our consciousness. The atmosphere or the environment gets into the individual (Brennan 2004: 1). Affect is not purely discursive, but discourse can mobilise and manipulate affect (Anderson 2016; McKenzie 2017). Firth (2016: 124) argues that ‘states can alter structures of affect through policy and discourse, and they do so to suit the needs of neoliberal capital’. Paying attention to affect can surface micropolitical subterranean tensions, pleasures and discomforts that are silenced in dominant policy discourses (Morley 1999). The political economy of neoliberalism in the late capitalist economy has been installed via material, discursive and affective means. It has forced a governing rationality and a globally circulating cluster of policy measures, involving deregulation and markets, and cultural regimes that privilege price and profit. There has been a re-articulation of measure and its relationship to value (Clough and Halley 2007). While this is all presented as a rational, objective and meritocratic process, it relies on a subterranean world of recognition, misrecognition, discrimination, inequalities and affect. Gregg and Seigworth (2010) claim that affect amounts to those visceral forces beneath, alongside or generally other than conscious knowing that can serve to drive us toward movement, thought and ever-changing forms of relation. Our assemblage approach affords a way of thinking about processes, practices, webs of relations, shifting subjectivities and feelings that can get stuck to mobility.

Internationalisation policy discourse is saturated in affect. It is embedded in policy formation including fear of missing out (FOMO), and imagined exclusionary futures, anxiety about catching and keeping up with the global ‘winners’ and trial by public exposure involves shame about private in-house matters being publicised in the public domain such as the global league tables. Fear of the (imagined) future is stimulated in the present via numerous crises discourses, disaster capitalism and the tendency to catastrophising. For example, we were informed that in the world of higher education, avalanches are on their way (Barber et al. 2013)^1^. In the midst of precarity and uncertainty, internationalisation, as a signifier of flexibility, flow and resilience, is represented as a happiness formula and a promise of the good life. Happiness and desire have become manipulated and integrated into the engines of the global knowledge economy (Ahmed 2010; Binkley 2014). An internationalised higher education system is presented as a Gestalt, a universalised and hegemonic system of determinants, with the whole being significantly greater than the sum of its isolated, fractured and peripheralised parts. On an individual basis, internationalising, or unsticking, one’s career is a pathway to success, signalling resilience and reach. Internationalisation deficit is a cause of shame implying stuckness and intellectual as well as spatial parochialism. Shame, according to Rose (1999), is central to the enforcement of norms. It is easily activated by the global arms race in higher education, particularly in relation to stasis and stuckness. While emotions can be unreliable targets of governmentality (Grant and Elizabeth 2015), they can produce and generate complex engagements of compliance and resistance.

## Higher education knowledge exchange and policy learning in the Asian century’

As part of a research project between the Centre for Higher Education and Equity Research (CHEER), University of Sussex, and the Research Institute of Japan, the UK and Europe (RIJUE), we gathered semi-structured interview data over two years (2017 and 2018) with thirty-four migrant academics (ten women and twenty-four men), and thirteen international doctoral researchers (five women and eight men). We constructed our sample to include English-speaking short-, medium- and long-stay migrant academics, with disciplinary locations in the humanities, social sciences and STEM (science, technology, engineering and mathematics). Our sample of short-stay doctoral researchers included five scientists, six social scientists and two from arts and humanities. We attempted to ensure a geographical spread. Twenty-five migrant academics were incoming from Australia, China, France, Germany, India, Nigeria, the Philippines, South Korea, the UK and the USA, and nine were Japanese who had worked in Canada, Germany, the UK and the USA. The doctoral researchers came from Bangladesh, China, Ghana, Nepal, the Philippines, South Korea, Syria, Taiwan, the USA and Vietnam. All participants have pseudonyms. We selected the universities to include a range of locations and institutional types e.g. fifteen private, five national and eight local public (municipal) universities. Our research team comprised CHEER academics and doctoral researchers working with Japanese academics in RIJUE to investigate how internationalisation is implemented, experienced, impeded or imagined in Japan, and to intersect mobility with issues of equity and affect.

Our research questions focused on why and how Japan was internationalising, and how migrant academics and doctoral researchers had experienced being international in Japan. Our interview questions interrogated divergent ways of encountering and understanding difference and included the following: what was driving the internationalisation policy agenda in Japan, how was it being implemented; personal, professional and academic motivations and experiences as migrant academics and doctoral researchers; and support and preparation. A consideration was what generates affective intensity, attunement and dissonance? We explored how participants navigate, inhabit and embody difference. Moments of affective intensity for our participants emerged in our data analysis in relation to coding narratives of the micropolitical challenges that they had experienced. These included the following: navigating gender regimes; contractual precarity, linguistic imperialism, orientalism and being ‘other’. We do not believe that there is an authentic subject buried beneath the overload of quantification that will surface given the ‘right’ questions, probes and cues, or that there is a ‘truth’ about academic mobility. But we do attempt to offer readings based on a call for a new ‘climatology’ of the social’ (Galloway and Thacker 2007). As Thrift (2008: 172) suggests, ‘issues like identity and belonging quiver with affective energy’.

## Navigating gender regimes: trailing spouses, bento boxes, token and targeted women

International mobility exposes the ‘stickiness’ of gender inequalities (Bernhagen 2017). Internationalising oneself can mean expressing and enacting affectivity in line with the demands and local specificities of the gendered affective arrangements. Significant global patterns in gendered power relations exist, but they also migrate, mutate and manifest differently across different spatial and social locations (Sang et al. 2013). Gender is a noun and a verb, and is about relational processes around particular types of social differentiation, as well as demographics. While there are substantial debates on post-binary gender (e.g. Hines 2018; Nestle et al. 2002; Nicholas 2014; Richards et al. 2017), and questions about whether we should even collect gender-disaggregated statistics as these reinforce gender binaries (Westbrook and Saperstein 2015), some gender-binaried statistics are troubling. For example, in Japan´s universities, there are four times as many male international faculty as females (Huang 2017). However, simple representation is not gender equality. We ‘do’ gender, and gender is intersected with a range of structures of inequality (Crenshaw 1991; Anthias et al. 1992). It counts in terms of who has the opportunity to internationalise and whether this interacts and sticks to gendered and classed opportunity structures and Global South/North power relations (Bhandari 2017; Jöns 2011; Matus and Talburt 2009; Myers and Griffin 2018; Rosner 2015). Internationalisation makes visible the embeddedness or stickiness of the patriarchal premium, and can serve to reinforce gender binaries by re-inscribing women and men in traditional gender roles. Leemann (2010) suggested that mobility is not viewed as a social experience whose value is neutral, but as something that has value precisely because it can be drawn into fields of asymmetrical gendered relations. She argued that women academics are less geographically mobile than their male counterparts, and that greater geographic immobility can put women at a disadvantage with regard to tenure.

Many of our participants expressed appreciation of the quotidian mobility that Japan offered women in terms of the safety of low crime rates. However, the unstuckness of the mobile subject can create stuckness for significant others. Our research found that one of the ways in which the gender premium manifests itself is through the heteronormative gender order of ‘trailing spouses’. Clarendon (2018) suggests that academic mobility offers three options for couples: split, go long-distance or sacrifice. As Skeggs (2010) noted, women’s labour (in its many permutations: care, parenting, domestic, affective) has been central to the reproduction of capital, but that it has been made invisible, surplus and naturalised, and is not counted in theories of value. We found examples of heterosexual male migrant academics supported by wives who had sacrificed their careers to follow their partners to Japan, or supported by Japanese wives who undertook all the cultural mediation and translation, childcare and homemaking for them. These arrangements fortified gender binaries. Women’s labour facilitated male professional mobility. While a surface reading suggested harmony, and cohesion, a closer analysis unearthed dissonance and affective intensity in relation to asymmetrical power relations. Sacrifice is part of the affective economy of austerity (Gill and Scharff 2011). Hans, a male German geologist, described how his wife’s doctoral education was sacrificed to support his mobility:

Now, I have one big downside…When we moved to New Zealand because I got a PhD scholarship, my wife was studying in Germany… And then in New Zealand, they changed a law … That meant, for us, all of a sudden that she would have to pay, I don’t know, $30,000 or something like this. We couldn’t afford it. That, basically, was the end for her career… From my experience with many other academics; one usually has to give up something.

Yi-ling, a Chinese doctoral researcher, outlined how her husband’s decision-making structured her choices:

I came to Japan, because he chose Japan as his destination... so that’s why I’m here now… my husband, his company is in Osaka. I have to stay in Osaka.

The women accepted ‘stuckness’ and stasis in their careers in order for their male partners to internationalise themselves professionally. However, wives’ stuckness and sacrifices had affective resonance, provoking discomfort and relational friction as Arjun, a male Indian Associate Professor scientist noted:

My wife is an engineer, IT engineer... She resigned purely because she got married to me … So, she gave up everything and then she did come here... She has two master's degrees…

Paradoxically, the fluidity of mobility strengthened the stickiness of heteronormative gender codes. Brett, a male history Professor from the USA, explained how he had attuned and benefitted from the gendered norm of homemaking for Japanese women:

We have three small kids, so she takes care of them…because there’s less pressure to get a job as a woman who’s taking care of small children over here. Maybe not as much these days, but there’s a certain acceptance of her role as a homemaker in this community but not as pronounced in the US, especially in New York City, where I used to live…. But over here… It’s not an exception or anomaly at all…It was really nice for me, actually.

Curt, an English language teacher from the USA in a private university, came to Japan with his Japanese wife who sacrificed her career, despite being in her home country:

She’s an accountant…I was working and financially we weren’t burdened, and we had a very small child, and eventually we had a second small child… about nine or ten months ago, she started talking about wanting to work, and I said, go ahead, but we compromised, that I didn’t want her to work full time. Also because of my job…She’s somewhat limited, too, in the number of hours she can work, because of my insurance^2^, I think, that she can’t work more than so many hours. Maybe it affects our taxes. … I know that she’s not doing workwise what she wants to do… I think if we were back home, she would be working full time, and she’s not doing that now, and I think even though she doesn’t express it, I do think it affects her, I think she would like to work and have more of a career, and she’s not having that right now*.*

While care work has always been an integral part of capitalist production, there is an affective economy linked to being a working mother in many national locations. Affective capitalism, gendered employment regimes and emotional cultures collided for Jane, an Associate Professor from the USA working in a private Japanese university:

People assume, still, that you can drop everything and come to the school for a conference or work in the bazaar or do things like that. … I’ve found it very hard to be a good teacher and a good administrator and a good researcher and a good mother. And when it comes to teaching and administration, those things you can’t, you can’t put them aside. So, the sacrifice has been for research.

Jane’s research career was sacrificed, and she had to navigate the frictions of frustration, socio-emotional contagion and loss in the identity collisions. Her actions exemplified Buchanan’s (2015: 383) observation that individuals develop their own ‘working arrangements’ in order to better cope with the demands of socially prevalent affective arrangements. However, these arrangements had repercussions for health, value production and gendered work performances.

Allison (1991) explored gender regimes in Japan via the socio-material expectation for Japanese mothers to provide aesthetically exquisite lunch (Bento) boxes for their children attending kindergarten. She argued that this was part of an ideological state apparatus that perpetuated patriarchy. Women were expected to live in relation to their families, not to be agentic and autonomous. These expectations create dissonance for those who wish to internationalise. Akiko, a late-career Japanese female science professor who eventually returned to Japan, explained that she moved to the UK as a young academic in order to unstick herself from Japan’s gender regime:

The second motivation why I decided to go abroad …my PhD supervisor told me it would be best for women to get married and stay at home. He even tried a marriage arrangement {Arranged marriage}…So I thought there would be no chance for me to find a good position to work…I thought there was no future for me in Japan, I wanted to go abroad.

Akiko’s current global status exemplified how micropolitics can operate as a driver for decision-making about mobilities, and how the micro and macro inter-relate, illustrating Horta and Yonezawa’s (2013) argument that Japanese academics that have gained doctorates abroad—for whatever reason—are more attuned to internationalisation.

The trailing, sacrificing wives’ discourse is saturated in binary gendered norms and heteronormativity, as many countries do not grant visas for same sex partners. LGBTQAI+ issues were rarely mentioned by our participants, with the exception of Jane who related how gayness stuck to foreign, rather than Japanese identities:

In Japan, 90 percent of gay and lesbian don’t come out… It’s considered to be a foreigner’s thing. It’s okay for us to be LGBT but Japanese say they can’t do it.

Heteronormativity and toxic masculinity contribute to gender regimes globally. Mai, a Vietnamese doctoral researcher in a Japanese national university, described how she had been subjected to sexual harassment from a male academic, and that no action had been taken:

My university had to do something for me because I have enough evidence, you know, he send messages to me, phone call, he came to my hostel and there are many, many things. They said you have enough evidence to report him and we can help you to get scholarship back but they didn’t do anything. They promised but they didn’t do.

In the neoliberal economy that constructs international students as a lucrative market, it is unsurprising (but completely unacceptable) that these matters get stuck and organisations globally frequently fail to investigate or acknowledge the misogyny, misconduct and sexual violence that make environments hostile and unsafe for women ‘consumers’. International students do not pay higher fees than domestic students in Japan (as in the UK), and many receive scholarships and bursaries. However, their financial value lies in the fact that they fill surplus higher education places, and this allows universities that have difficulties filling quotas to gain government subsidies, and indeed, to survive. Female international students globally can be orientalised targets for predatory behaviour as they are conceptualised as reluctant to speak back to power for cultural and material reasons. While many universities globally have policies on sexual violence, it has often been left to the media to expose these pervasive patriarchal practices (Busby 2018; SBS News 2018). Feminist theorists have argued that vulnerability discourse distracts by focusing on survivors (Butler et al. 2016; Mählck 2018). Identifying sexual violence and misogyny can involve an affective trajectory or process of de-naturalising it—consciousness-raising, and attributing it to patriarchal cultures, rather than to the transgressions of a few disturbed perpetrators. This is a challenging task for international women who can be dismissed as misrecognising/misinterpreting and being non-attuned to local social interactions.

The recruitment of female migrant academics was perceived by some as an intervention to shift Japan’s gender statistics. The quantitative under-representation of women, and its associated tokenism was commented on by Chen, a female Chinese sociology Professor in a private university:

I think Japanese academia, as you probably have noticed, is very male. (My) University has about 10 percent female faculty…There is a very big gap between the student demographic profile and the faculty profile… I think the promotion is less, as I think it is worldwide, right? … Women academics advance slower than men.…And (my university) selected ten so-called next generation core researchers, basically researchers in their 40s who they feel are promising…I’m the only woman… I think sometimes you can’t shake the feeling, am I the poster child…the token…am I the designated woman researcher? … I think as a woman, as a foreigner, sometimes it might be irrational and it might be paranoia, you do think, well, is it because of my profile, and either for good or for bad?

The ecology of material and affective unpredictability meant that even when women were winning, they felt that they were losing—a highly precarious ontological position.

## Punishing Precarity

The political economy of neoliberalism can micropolitically produce fear and anxiety partly in response to precarity, performance measurement and cognitive capitalism (IPC 2014; Shahjahan 2019). Precarity interacts with gender and other structures of inequality (Read and Leathwood 2020). Affective intensity is exacerbated when one is negotiating contracts, visas and employment and study regimes in a country different from the homeland. Last-minute modality and the just-in-time economy mean that precarity is ‘an ontological experience and social-economic condition’ (Neilson and Rossiter 2008:55). It relates not only to the risks of contractual arrangements, but also to an affective or existential state, understood at once as a source of ‘political subjection, of economic exploitation and of opportunities to be grasped’ (Lazzarato 2004). Precarity has been described as a form of ‘embodied capitalism’ (Tsianos and Papadopoulos 2006). Migrant academics, as non-citizens (Japan does not allow dual citizenship), can be positioned as precarious, contingent or flexible workers, with the dispositions to unstick themselves unproblematically. In a Faustian pact, they have traded security for adventure. Internationalisation offers a coexistence of contradictions. Migrants are simultaneously privileged (especially if they are white, heterosexual, male Anglophone from the Global North), vulnerable and susceptible to injury (Butler 2004), with identities located between calculative choice and victims of geopolitical and socio-economic flux, flexible accumulation and uncertainty. They are both detached and affectively invested. As with most competitive employment regimes, success is available to some and foreclosed for others. Scott, a Humanities Associate Professor from the USA, described how his employment contract was a potent affective object and socio-material practice in his private university. He found himself stuck and dependent on the fluid nature of the informal to transform his outsider into insider subjectivity:

And then over time I’ve gotten a couple of contracts. … my first was a five-year contract, and then a three-year contract. And then they ran out of contracts. And you know, that’s how it works in Japan, if they run out of contracts, then they say goodbye, right?… I was really stressed, and I felt, wow, you know, if I could have cut and run, and gone somewhere else, so…Well, it was weird… some faculty member that I had never really talked to before, walked by me, and smiled and said, ‘I think we found something for you’. And I was like, you know, ‘what’s going on here?’ … It actually felt like I was joining the mob in the someway.

The uncertainty is evocative of Berlant’s (2011) concept of cruel optimism—when something you desire is actually an obstacle to your flourishing as you are unlikely to achieve it and the wanting makes you more governmentable. Precarity, according to Neilson and Rossiter (2008:64), becomes an experience from which ‘differential capacities and regimes of value emerge’. Value production means that some bodies were more vulnerable to institutional and social misrecognition, it appears. Scott’s identity was re-cast/unstuck from disposable outsider to valued insider via an overnight ad hoc change in contractual status. The identity premium that stuck to him of being a white male Anglophone US citizen allowed him to convert capital into value, but it also demanded gratitude and an increase in indebtedness, or stickiness (Firth 2016). Berlant’s analysis of the affective conditions of precarity suggest that they can result in a micropolitical ‘aspirational normativity’—the state of trying to construct ‘a less-bad bad life’ (2007: 291). Any contract is better than no contract at all.

Precarity was also experienced in relation to differential services and interactions. Differences in approaches to doctoral supervision between international and home students were cited as being relatively common, as Aasha, a female Nepalese doctoral researcher, explained:

They [Japanese doctoral researchers] come sometimes to the lab and are absent for weeks and again come, few or not all, and we international students and also private students^3^, we need to go there every day. But still if some students delay on their work or updating their work, sensei (supervisor) …scolds them. But Japanese students …We never notice him scolding them.

Differentiation was a key theme in our research (Arudou n.d.). Pierre, a French citizen and history Lecturer, discussed the different contracts for Japanese and non-Japanese academics in his university:

So, in our university we still have non-Japanese contracts. I mean, our contracts are especially for us. We have a different regime, different system… non-Japanese people, we only get five years’ contract.

Racism is a global, not a local problem, however, it was highly visible to some of our participants who had migrated from societies where legislation existed to regulate its most extreme forms of expression. John, an English Language lecturer from the UK, observed:

Regarding gender and race, Japan can be a little bit traditional should I say… There was a study of an interesting experiment done about hiring in Japan, where they sent a resume, and in Japan you have to show a photograph, so they sent a black candidate’s resume around Japanese university hiring. And this person had a very good resume. It was a made-up resume because they were doing an experiment. And then they sent a white, Caucasian, blond hair, blue- eyed resume with less experience and fewer publications … And that person got shortlisted and the other person didn’t, and that’s worrying in terms of hiring.

Misrecognition was embedded in migrant academics’ reports of being subjected to commodity logic; recruited not for their epistemic value and disciplinary knowledge, but rather to teach English—either as a foreign language to Japanese students and staff, or to teach programmes in English (EMI: English as a medium of instruction) to attract more international students and hence augment the internationalisation data for their universities. Commodification was also experienced micropolitically and reflected on by the doctoral researchers, as Myung, a Korean male doctoral researcher in the social sciences, stated:

Sometimes I feel like they want to get more international students because of their reputation or their evaluation done by the Government.

In neoliberal audit regimes, the presence of international bodies indicated positive attunement with the global knowledge economy.

## Deep and surface internationalisation: joining the global community or linguistic imperialism?

Language was a site of affective intensity in our study—the promotion of the English language as a signifier of internationalisation, the linguistic privilege of the migrant Anglophone communities and the challenges of being a non-Japanese speaker (Hashimoto 2007; Stewart and Miyahara 2011). Our research found that many migrant academics were of material and symbolic value to Japanese universities, as so many positive attributes stuck to the English language. They taught programmes in English (EMI), taught English as a foreign language and augmented the diversity statistics, but were often excluded from epistemic communities. Futoshi, a male Professor in a national university, berated the surface internationalisation that stuck to language:

Until 2010, almost zero of our courses were taught in English…This year, 120 courses were taught in English. So, we’ve improved a lot…Englishinisation, Americanisation, and Anglo-Saxon kind of model, it’s not internationalisation… But the government is really pushing degree programmes in English. They’re promoting, they’re giving money.

Tension existed between policy aspirations to internationalise and micropolitical resistance to linguistic imperialism (Morley et al. 2019). Gottlieb (2005: 75) also describes a ‘tension between two arms of internationalisation policy—teaching English and promoting the spread of Japanese’. The English language was experienced as a devouring monster that threatened local cultures and the Japanese language was perceived as too regionalized to justify investment by short-term migrants. Futoshi discussed this binary of the English v Japanese languages:

I always encourage students to learn Japanese. ‘Professor, I feel I don’t have to speak Japanese here. I don’t learn Japanese here because too many vending machines. Without speaking I can buy the things. You go to the convenience store without speaking, the change is always correct. There’s no struggle. There’s no problem here. You don’t have to negotiate with anything. Why do I have to learn Japanese?’ ‘No, you have to make Japanese friends’, that’s what I’m saying.

The linguistic binary meant that all foreigners were expected to speak English and Japanese colleagues spoke Japanese. Mai suggested that Japanese colleagues were not attuned to speaking English:

Some people I know they speak English well. Even some Professors can speak English very well but they don’t speak English to students.

One explanation for resistance to the English language was honour and perfectionism. Toh (2016) recognised this cultural concept of *hazukashigaru* (embarrassment) in relation to speaking English, even when individuals had a high level of proficiency.

Catch-up is part of the logic of the Asian Century (West 2018). Unlike in the former British colonies of Singapore and Hong Kong, this includes anxiety about English proficiency in Japan. In 2017, Japan’s average total score on the TOEFL was 71, the second lowest in Asia (Education Testing Service 2018). Japan’s once successful economy can no longer insulate them from the linguistic demands of globalisation. The affective intensity of ‘catch- up’ facilitates the recruitment of academics for their language rather than discipline. Some participants—especially those teaching English—described how they were ‘othered’, ghettoised or instrumentalised as agents of the confected internationalisation of Japanese students and staff. They believed that their professional development and advancement needs were overlooked and they complained of being stuck in a space of overwork, marginalisation and social and epistemic exclusion. John outlined his sense of entrapment:

I have huge frustrations in my job. On one hand I’m given a lot of freedom to do things, but on the other hand I’m excluded from certain committees that I feel I should be on… I’ve hit a glass ceiling now…At the institutional level, at the professional level there’s still so much that I can achieve and do, so that’s where I focus my attention. But considering that I’ve got 20 years’ experience of language teaching, I’m internationally published, I write materials for international publishers and I do teaching training. I’ve been around China, Indonesia and places like that doing teacher training. And yet my own institution doesn’t utilise my skills and keeps me marginalised, is one of my biggest frustrations.

Being stuck in monolingualism meant that international doctoral researchers felt that they were excluded from research opportunities, as Nicole, a female doctoral researcher in Management from the Philippines, commented:

Again, I’m sorry, but I keep going back to the language. It was mostly because a lot of information was all in Japanese, such that I felt that perhaps certain opportunities you miss because of that.

The above narratives indicate how difference frequently sticks to language. In the sensuous social order foreigners are interpellated as other, strangers who are not attuned or aligned with the local collective affects, and are subjected to a perpetual tribunal of assessment. The cosmopolitan deracinated intellectual is a citizen of nowhere (Rose 2017). Being ‘other’ holds up a mirror that reflects both the local cultures and the identities and patterns of the migrants. Discomfort and dissonance become naturalised. Socio-political power geometries are reduced to micropolitical, private problems of etiquette, linguistic competence and the re-ordering of one’s affective life.

## Concluding comments

Transnational encounters involve a politics of translation (Pedwell 2014). While internationalisation offers transformative professional and rich personal experiences, it can also reproduce dominant social and geopolitical hierarchies, regimes and exclusions. Critics of Japan’s internationalisation processes argue that it is nationalism under another name e.g. Japan’s ambition to rise to a position of singular importance and power in the global knowledge economy (Rivers 2010; Yonezawa et al. 2009). Paradoxically, becoming unstuck leads to further stickiness. Hashimoto (2000:45) suggests that ‘the promotion of internationalisation seems to aim to re-educate Japanese citizens to reassert their collective identity as Japanese’. Internationalisation, for Burgess (2010: 10), ‘is less about transcending cultural barriers and more about protecting them’. These tensions might account for some of the micropolitical discomforts, and binary logic of ‘us’ and ‘them’ expressed in our participants’ affective assemblages. It could also reflect a Japanese cultural concept of *uchi* (inside) and *soto* (outside) (Lebra 1976) 4. Our affective readings of the narratives of migrant academics and doctoral researchers are not a truth, but a way in which to explore the mobility/power conjunction in so far as they highlight how internationalisation is differentially felt, resisted, imagined, mediated, negotiated and desired. This is not unique to Japan (Morley et al. 2018). Mobility suggests a linguistic antithesis to stuckness, but provides new forms of stickiness and entrapment and an affective infrastructure that is disqualified from discourse e.g. the precarity of short-term contracts, ‘outsider’ identity challenges enacted in language and culture and the requirement to adapt to traditional/conventional gender regimes. On the one hand, mobility symbolises a new, enriched, post-national cosmopolitanism for individuals, knowledge and higher education systems. However, it could also represent a form of disposability and deletion in the accelerated and highly instrumental and commodifying market economy of the neoliberal global academy necessitating significant affective and gendered labour in mediating the geopolitics of knowledge.

## Footnotes

1 This research was conducted before the global Covid-19 pandemic of 2019–2020—a crisis that will have a significant impact on internationalisation, mobility and spatiality in higher education.

2 In Japan, if a spouse’s annual income is less than 1.2 million, the ‘breadwinner’ can receive an allowance for a spouse from his or her employer. In reality, the tax system encourages women to stay home as homemakers.

3 There are two types of international students in Japan. One is scholarship students, and the other is privately financed students.

4 See http://japanology.org/2017/03/concept-of-uchi-soto-in-groups-and-out-groups/
